# Impact of hindcast length on estimates of seasonal climate predictability

**DOI:** 10.1002/2014GL062829

**Published:** 2015-03-12

**Authors:** W Shi, N Schaller, D MacLeod, T N Palmer, A Weisheimer

**Affiliations:** 1 Department of Physics, Atmospheric, Oceanic and Planetary Physics, University of OxfordOxford, UK; 2 Department of Physics, National Centre for Atmospheric Science (NCAS), University of OxfordOxford, UK; 3 European Centre for Medium-Range Weather ForecastsReading, UK

**Keywords:** predictability

## Abstract

It has recently been argued that single-model seasonal forecast ensembles are overdispersive, implying that the real world is more predictable than indicated by estimates of so-called perfect model predictability, particularly over the North Atlantic. However, such estimates are based on relatively short forecast data sets comprising just 20 years of seasonal predictions. Here we study longer 40 year seasonal forecast data sets from multimodel seasonal forecast ensemble projects and show that sampling uncertainty due to the length of the hindcast periods is large. The skill of forecasting the North Atlantic Oscillation during winter varies within the 40 year data sets with high levels of skill found for some subperiods. It is demonstrated that while 20 year estimates of seasonal reliability can show evidence of overdispersive behavior, the 40 year estimates are more stable and show no evidence of overdispersion. Instead, the predominant feature on these longer time scales is underdispersion, particularly in the tropics.

**Key Points:**

- Predictions can appear overdispersive due to hindcast length sampling error
- Longer hindcasts are more robust and underdispersive, especially in the tropics
- Twenty hindcasts are an inadequate sample size to assess seasonal forecast skill

## 1. Introduction

There is no question that skillful seasonal forecasts can be made in the tropics [e.g., *Barnston et al.*, 2012]. However, the extent to which seasonal forecasts have useful information in the extratropics is more controversial. For example, while on the one hand *Scaife et al.* [2014] recently showed that the new UK Met Office seasonal forecast model Global Seasonal forecast system version 5 (GloSea5) was able to skillfully predict the wintertime North Atlantic Oscillation (NAO) index for the period 1993–2012, on the other hand, *Weisheimer and Palmer* [2014] demonstrated that seasonal predictions of temperature and precipitation were not reliable for several regions in the extratropics, in particular over Europe.

Using the data set of skilful NAO forecasts in GloSea5, *Eade et al.* [2014] suggested that seasonal forecast ensembles created with initial condition uncertainty were underconfident, or overdispersive, with too much noise in each ensemble. These would lead to an unreasonably pessimistic estimate of seasonal predictability because the potential skill would be underestimated implying that the real world would be more predictable than the model world.

One of the difficulties with seasonal prediction research is that the sample size from which estimates of forecast skill can be obtained is necessarily small: for start dates at a given time of year, the sample size of seasonal forecasts for boreal winter from the 20 year period 1992–2011, as used in *Eade et al.* [2014] is just 20. For example, one would hardly implement changes to a numerical weather forecast model based on a sample of just 20 forecasts. Indications that 20 may be too small a number for robust estimates of skill can be found in studies, e.g., by *Müller et al.* [2005] who showed that robust results for the seasonal forecast skill of the NAO index were not stable with a sample size of 20 and by *Kumar* [2009] demonstrating the effect on skill measures of small verification time series due to sampling error.

In this paper we assess the *Eade et al.* [2014] claim that model estimates of extratropical predictability in the North Atlantic region are unduly pessimistic. We analyze a consistent set of seasonal forecast ensembles from a total of eight individual models over various subset of the 42 year period 1961–2001. It is found that several of these models have NAO hindcast skill levels comparable to GloSea5. We then compute the “Ratio of Predictable Components” (RPC) diagnostic of *Eade et al.* [2014] to show that while individual model ensembles can appear overdispersive over 20 year periods, they are not overdispersive over 40 year periods.

We conclude that the claims made in *Eade et al.* [2014] are consistent with sampling uncertainty due to the limited length of the hindcast period. However, on 40 year timescales, evidence suggests that single-model ensembles are profoundly underdispersive. This suggests that it remains crucially important to develop reliable methods to represent parametrization uncertainty [*Palmer*, 2012; *Weisheimer et al.*, 2014].

## 2. Methodology

We use seasonal hindcast simulations over a 42 year period performed with eight individual model ensembles as part of in the European Union projects *DEMETER* [*Palmer et al.*, 2004] and *ENSEMBLES* [*Weisheimer et al.*, 2009] to revisit the findings of *Eade et al.* [2014] and to asses the predictability of the NAO. As is well known [*Hurrell et al.*, 2001], the NAO is a mode of atmospheric variability over the North Atlantic region with wide-ranging impacts on the weather and climate over Europe. In this paper, a simple index of the NAO is defined following *Pavan and Doblas-Reyes* [2000] and *Doblas-Reyes et al.* [2003], taking the model projections of the forecast anomalies of geopotential height at 500 hPa (Z500) on the leading climatological Empirical Orthogonal Function. In addition, we also computed an NAO index based on the normalized mean sea level pressure (MSLP) difference between the Azores and Iceland.

The following models were used in our analysis: *D_ECMF* (European Centre for Medium-Range Weather Forecasts (ECMWF)), *D_UKMO* (MetOffice), *D_MEFR* (MétéoFrance) from the DEMETER system and *E_ECMF* (ECMWF), *E_UKMO* (MetOffice), *E_KIEL* (IfM Kiel), *E_INGV* (INGV Bologna), and *E_MEFR* (MétéoFrance) from the ENSEMBLES system. The individual model ensembles consist of nine members that were created through perturbed initial conditions. For the analysis we consider seasonal mean forecast anomalies for December to February (DJF) from forecasts started on 1 November each year. The verification data were obtained from the ERA-40 Reanalysis Project [*Uppala et al.*, 2005].

Following *Eade et al.* [2014], ensemble-based estimates of predictability can be obtained from a diagnostic known as the Ratio of Predictable Components (RPC) between the observed and model predicted values defined as


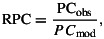


where PC is the predictable component in observations and in model hindcasts. In *Eade et al.* [2014], this is approximated by


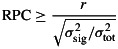


where *PC*_obs_ is estimated directly from the explained variance given by the square of the correlation coefficient *r* between the ensemble mean model forecasts and the observations. The authors used the variance of the ensemble mean (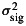) relative to the average variance of individual ensemble members (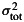) to estimate *PC*_mod_. For a perfect forecast system, the *RPC* should be close to 1. RPC values greater than one imply that the model is unduly pessimistic in its estimate of skill, by being overdispersive. Conversely, RPC values below 1 point toward underdispersive and overconfident forecasts.

However, such an interpretation has limitations. As discussed in *Kumar et al.* [2014], the definition of the model predictable component PC_mod_ depends on the particular forecast model used and cannot necessarily be indicative of the *true* potential predictability. Differences between actual skill levels (or *PC*_obs_ as estimated through the correlation *r* between the ensemble mean and observation) and potential skill of a perfect model (or PC_mod_ as estimated through the average correlation *r*_perf_ between the ensemble mean and the individual ensemble members) are related to errors in the model that lead to imperfect biased forecasts. Furthermore, the above interpretation is only valid with a sufficiently large hindcast length and ensemble size. With an insufficient sample size, estimates of *RPC* can fluctuate above or below unity purely by chance, and no physical conclusions can be reached about whether the ensemble system is underdispersive or overdispersive overall.

Here we analyze the RPC of the NAO index and, similar to *Eade et al.* [2014], the global MSLP fields simulated by the individual DEMETER and ENSEMBLES hindcasts. In order to study the impact of the hindcast length on NAO skill and RPC, we analyze a large number of combinations of hindcast years based on the full hindcast period 1960–2001. Combinations of hindcast years were generated by randomly and independently sampling from the very large number of all possible combinations of 5,10,15…40 years out of the total 42 year period. For example, there exist 861 possible combinations of randomly sampled 40 years. For shorter subperiods there exist more conceivable combinations with a maximum of more than 500 billion possible combinations for 20 year periods. In order to have a comparable sample size for all considered subperiods, our results are based on 20,000 draws from the combinations, with repetition.

We have tested the sensitivity and robustness of our results for longer hindcast periods using two approaches: The first approach involved modifications of the random draws of hindcast years by allowing resampling of years in each draw (with replacement). The second approach is based on the finding that the maxima of the RPC distributions for shorter hindcast periods up to 20 years can be approximated very well by an exponential decay function, dependent on hindcast length. These exponential fits in turn provide an alternative tool to extrapolate the RPC maxima for hindcast periods longer than 20 years. While both approaches were found to result in some minor differences as to the exact shape of the RPC distributions for long hindcast periods (not shown), the uncertainty ranges from our resampling methodology as outlined above are consistent with these estimates.

## 3. Results

The NAO correlation coefficients between the ensemble mean and the verification data for three different hindcast periods are given in Table 1 for the DEMETER and ENSEMBLES individual models for both the Z500-based and MSLP-based definitions of the NAO index. Consistent with the results of *Müller et al.* [2005] it shows that there are differences in the level of predictive skill between the two shorter subperiods. This itself is indicative that a 20 year period may be insufficient for a robust estimation of overall predictive skill. The correlation between the modeled NAO indices and observations tends to be higher for the late period 1980–2001 than for the early period 1960–1979. Some of the individual models show significant correlations for the 20 year subperiods (0.59 for *D_MEFR*, 0.45 for *D_ECMF*, and 0.60 for *E_KIEL*). These levels of skill are comparable with the values reported in *Scaife et al.* [2014]. However, when we look at the entire 42 year hindcast period 1960–2001, the correlations are considerably lower.

**Table 1:** NAO Correlations Between Model Ensemble Mean and Observations Based on Z500 (MSLP) for Different Hindcast Periods^a^

	*E*_*ECMF*	*E*_*UKMO*	*E*_*KIEL*	*E*_*INGV*	*E*_*MEFR*
1960–1979	−0.16 (−0.35)	0.03 (0.17)	0.12 (**0.60**)	0.03 (−0.39)	0.07 (0.19)
1980–2001	0.20 (0.35)	0.02 (−0.08)	−0.07 (0.11)	0.22 (0.30)	0.35 (0.33)
1960–2001	0.07 (0.08)	−0.02 (0.00)	−0.08 (0.26)	0.10 (−0.02)	0.21 (0.26)
	*D*_*MEFR*	*D*_*ECMF*	*D*_*UKMO*		

1960–1979	0.26 (0.35)	−0.42 (0.10)	−0.05 (**−0.47**)		
1980–2001	**0.59** (0.32)	**0.45** (−0.05)	0.21 (0.02)		
1960–2001	**0.38** (0.20)	−0.12 (−0.06)	−0.15 (−0.27)		

a The first part shows results from the ENSEMBLES models. The second part shows results from the DEMETER models. Correlations where a *t* test suggests significance at the 95% level are marked in bold.

**Figure 1:**
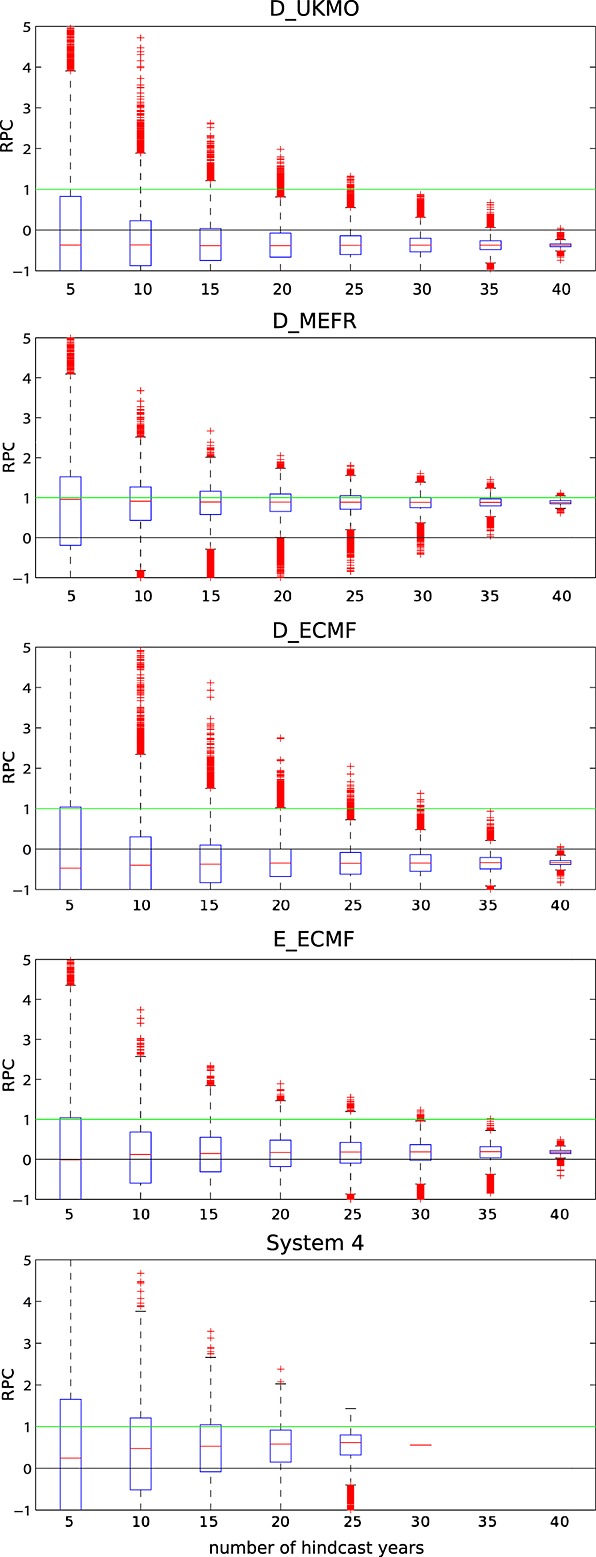
Box-and-whisker plots of the distributions of RPC of the Z500-based NAO index in DJF as a function of the number of hindcast years used in (a–c) the three DEMETER models, (d) the ENSEMBLES model of ECMWF, and (e) ECMWF's currently operational forecasting system 4. The box includes 50% of the data and the whiskers indicate approximately the 99% and 1% percentiles of the distribution. Outliers are marked with crosses.

From the above described sampling algorithm, distributions of RPC values for the NAO index (Z500 and MSLP) have been derived. Figure 1 shows these distributions for the Z500-based index as box-and-whisker plots from the three DEMETER models *D_MEFR*, *D_UKMO*, and *D_ECMF* together with the two more recent versions of the ECMWF seasonal forecast model: the version used in ENSEMBLES (identical to ECMWF's System 3) and the currently operational System 4 (for which only 30 years of hindcast data exist, see also *Stockdale et al.* [2015]). For each ensemble the RPC distributions for different lengths of hindcast data between 5 and 40 years (25 years in the case of System 4) are displayed. As the length of the hindcast period increases, the RPC values for all models decrease and the spread narrows. For the 5 year period, the RPC range includes both large negative and large positive values. For the 20 year period, in particular, the upper range of the RPC still clearly exceeds values of 1. Qualitatively very similar behavior was found for the analysis using either the MSLP-based NAO index or the other ENSEMBLES models, see figures in the supporting information.

Since present-day operational seasonal forecast models are likely to be more skillful than the typical ENSEMBLES models, as is the case of GloSea5, the upper range of the RPC distribution will be more representative of possible RPC values from contemporary models. However, when 40 years of data are considered, no single model except for one gives RPC values above 1 (the upper whisker of the distribution for *D*_*MEFR* just reaches 1). Indeed, the entire distribution of RPC values for 40 years lies below 1 for all but one model.

**Figure 2:**
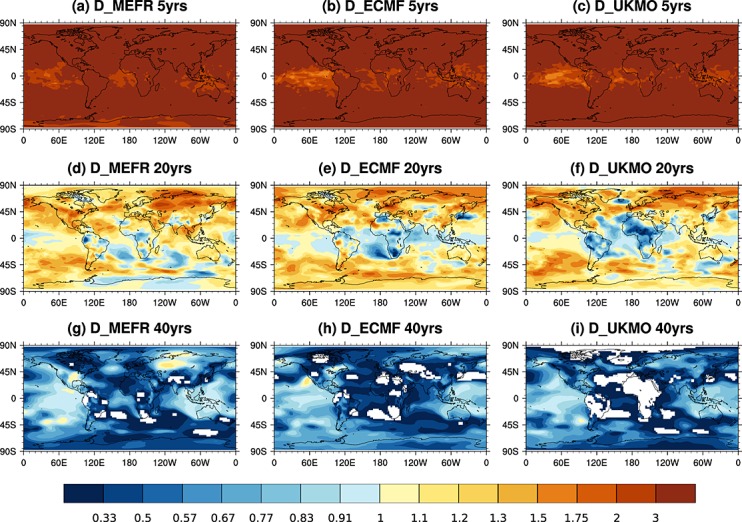
Maximum value of each RPC grid point distribution for mean sea level pressure in DJF. Results for 5 year, 20 year, and 40 year time periods are shown for the three individual DEMETER model ensembles. Regions of negative RPC are masked out in white.

Following *Eade et al.* [2014], Figure 2 shows global maps of RPC for mean sea level pressure forecasts in DJF. The value of RPC shown is the maximum RPC for each grid point distribution based on 5000 samples of possible combinations of hindcast years. For clarity we only show the three individual DEMETER models for subperiods of 5 (Figures 2a–2c), 20 (Figures 2d–2f), and 40 (Figures 2g–2i) hindcast years. The corresponding figures for the ENSEMBLES models can be found in the supporting information. For a 5 year sampling period, the maximum RPC is above 1 everywhere. When 20 years of hindcast data are available, the maximum RPC in general decreases, with the tropics already indicating values below 1. At 40 years, most of the regions of the world have maximum RPC values that fall below 1. Contrary to *Eade et al.* [2014] this shows that when a sufficiently long hindcast period is used so that the distribution of RPC converges, the seasonal forecast ensembles from individual models are not underconfident (overdispersive) but rather overconfident (underdispersive), in particular in the tropics.

## 4. Summary and Conclusion

In this study, we have investigated the seasonal forecast NAO skill during DJF in terms of correlation between the ensemble mean and observations for a variety of seasonal forecast models of the DEMETER and ENSEMBLES projects over different time periods. For 20 year hindcast periods significant correlations for the NAO index were found, in agreement with the results presented in *Scaife et al.* [2014]. However, no model produced significant correlations throughout the entire 42 year period where seasonal hindcasts were available (1960–2001).

In addition, we have analyzed the Ratio of Predictable Components (RPC) for seasonal hindcasts of the NAO and mean sea level pressure in DJF. For periods of 20 years or less, the distribution of possible RPC includes values greater than one which indicate overdispersive conditions. However, for periods of 40 years, the maximum of all the distributions of RPC is always less than 1. This implies that the ensembles, if evaluated on longer time scales, are not overdispersive. Indeed, by studying global surface pressure, the overriding problem with single-model ensembles is their underdispersiveness in the tropics. The interpretation of our results can lead to the conclusions that the findings of *Eade et al.* [2014] merely suggest an inadequately small sample size where extreme values of the RPC can easily be found above 1 when a 20 year sample size is used. These extremes, however, all fall to values below 1 for the tested model hindcasts if a 40 year hindcast period is used in the statistical analysis. Our results suggest that increasing the sample size of GloSea5 by extending its current seasonal hindcasts length back to the 1960s would enable to test the robustness of GloSea5's dispersion behavior on longer time scales.

Although our results suggest that single-model ensembles are not overdispersive on average, it is still possible that current-generation climate models simulate a smaller range of predictability than does the real world. This might occur if the real climate attractor is more heterogeneous than the model attractor. That is to say, the real-world attractor may have more distinct regions of stability and instability than does the more diffusive climate model attractor. Hence, when the real world is evolving in a region of strong predictability, the ensemble may be overdispersed and hence underconfident. Conversely, when the real world is evolving in a region of weak predictability, the ensemble may be underdispersed and hence overconfident. This is consistent with the notion that state-space probability distributions for the real atmosphere show evidence of quasi-stationary regimes, while simulated probability distributions, especially from low-resolution climate models, tend to appear overly Gaussian [*Dawson et al.*, 2012].

The analysis of *Eade et al.* [2014] also studied ensemble forecasts from decadal prediction experiments over the 46 year period 1960–2005. They concluded that the RPC for mean sea level pressure over the globe is also underconfident (overdispersive), similar to the seasonal forecast ensembles. However, the analysis is based on 4 year averages of sea level pressure where the forecasts starting every year overlap in time. This implies a large degree of dependence between the individual forecasts as there is considerate overlap between the target forecast periods from different start years. Thus, the effective independent sample size of the hindcasts is not 46 but rather of the order of 10. By analogy, a similar situation would arise if one wanted to forecast the winter anomalies from seasonal forecasts pooled together from several months of start dates across the autumn and early winter of a given year; these forecasts cannot be counted as independent samples.

The number of members in the forecast ensemble is another source of sampling uncertainty when estimating the correlation skill and RPC. While this study is focused on the effect of the hindcast length, work to analyze how the ensemble size influences these estimates is under way.
