# Supplementary material for: Impact of hindcast length on estimates of seasonal climate predictability
Source: Geophys Res Lett. 2015 Mar 12;42(5):1554–9. doi: 10.1002/2014GL062829 (PMC4459196; doi:10.1002/2014GL062829)
Supplement: Supplementary file 1 — Figures S1–S5 [file grl0042-1554-sd1.doc]

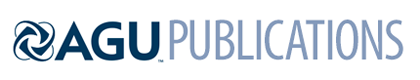


*Geophysical Research Letters*

Supporting Information for

**Impact of hindcast length on estimates of seasonal climate**

**predictability**

Shi, W.^1^, N. Schaller^1^, D. MacLeod^1^, T.N. Palmer^1,2,3^ and A. Weisheimer^1,2,3^

^1^Department of Physics, Atmospheric, Oceanic and Planetary Physics, University of Oxford, Oxford, OX1 3PU, UK

^2^Department of Physics, National Centre for Atmospheric Science (NCAS), University of Oxford, Oxford OX1 3PU, UK

^3^European Centre for Medium-Range Weather Forecasts (ECMWF), Reading, UK

**Contents of this file**

Figures S1 to S5

**Introduction**

This supporting information consists of 5 figures which complement the article.

Figure S1. Distribution of RPC values of the NAO index based on MSLP for all 3 *DEMETER* models.

**Figure S2.** Distribution of RPC values of the NAO index based on Z500 for all 5 *ENSEMBLES* models.

**Figure S3.** Distribution of RPC values of the NAO index based on MSLP for all 5 *ENSEMBLES* models.

Figure S4. **Distribution of RPC values of the NAO index based on MSLP for System 4.**

Figure S5. Global maps of maximum RPC values of MSLP for all 5 *ENSEMBLES* models for hindcast periods of 5, 20 and 40 years.
